# Testosterone induces sexual dimorphism during infection with *Plasmodium berghei* ANKA

**DOI:** 10.3389/fcimb.2022.968325

**Published:** 2022-09-27

**Authors:** Jesús Aguilar-Castro, Luis Antonio Cervantes-Candelas, Fidel Orlando Buendía-González, Omar Fernández-Rivera, Teresita de Jesús Nolasco-Pérez, Monserrat Sofía López-Padilla, David Roberto Chavira-Ramírez, Armando Cervantes-Sandoval, Martha Legorreta-Herrera

**Affiliations:** ^1^ Unidad de Investigación Química Computacional, Síntesis y Farmacología de Moléculas de Interés Biológico. Laboratorio de Inmunología Molecular, Facultad de Estudios Superiores Zaragoza, Universidad Nacional Autónoma de México (UNAM), Ciudad de México, Mexico; ^2^ Posgrado en Ciencias Biológicas, Universidad Nacional Autónoma de México, Ciudad de México, Mexico; ^3^ Departamento de Biología de la Reproducción, Instituto Nacional de Ciencias Médicas y Nutrición Salvador Zubirán, Ciudad de México, Mexico; ^4^ Laboratorio de Aplicaciones Computacionales, Facultad de Estudios Superiores Zaragoza, UNAM, Ciudad de México, Mexico

**Keywords:** malaria, immune response, sexual dimorphism, testosterone, *Plasmodium berghei* ANKA

## Abstract

Malaria is the most lethal parasitic disease worldwide; men exhibit higher mortality and more severe symptomatology than women; however, in most studies of immune response in malaria, sex is not considered a variable. Sex hormones 17β-oestradiol and testosterone are responsible for the main physiological differences between sexes. When interacting with their receptors on different immune cells, they modify the expression of genes that modulate cell proliferation, differentiation, and synthesis of cytokines. The immunosuppressive activity of testosterone is well accepted; however, its participation in the sexual dimorphism of the immune response to malaria has not been studied. In this work, we analysed whether altering the concentration of testosterone, through increasing the concentration of this hormone for exogenous administration for three weeks, or gonadectomy before infection with *Plasmodium berghei* ANKA affects different cells of the immune response necessary for parasite clearance. We also assessed the concentration of pro-and anti-inflammatory cytokines in male and female CBA/Ca mice infected or not with the parasite. Our results show that testosterone changes affect females more than males, resulting in sex-associated patterns. Testosterone administration increased parasitaemia in intact males while reducing it in intact females leading to a dimorphic pattern. In addition, gonadectomy increased parasitaemia in both sexes. Moreover, testosterone administration prevented both weight loss caused by the infection in females and hypothermia in gonadectomized mice of both sexes. Boosting testosterone concentration increased CD3^+^ and CD8^+^ populations but decreased the B220^+^ cells exclusively in females. Additionally, testosterone reduced IFN-γ concentration and increased IL-6 levels only in females, while in males, testosterone increased the number of NK cells. Finally, gonadectomy decreased TNF-α concentration in both sexes. Our results demonstrate that testosterone induces different patterns depending on sex and testosterone concentration. The results of this work contribute to understanding the impact of modifying testosterone concentration on the immune response specific against *Plasmodium* and the participation of this hormone in sexual dimorphism in malaria.

## Introduction

Malaria is the most lethal parasitic disease in the world and causes fever, anaemia, weight loss, hepatomegaly, splenomegaly and, in the most severe cases, death (WHO, 2021). It exhibits a marked sexual dimorphism, with males showing higher parasitaemia, symptom severity and mortality than females. Sex hormones are involved in the sexual dimorphism of the immune response in malaria (Lopes et al., 2016; Legorreta-Herrera et al., 2018; Cervantes-Candelas et al., 2021); oestrogens induce immunocompetence. In contrast, testosterone, the primary male hormone, generates immunosuppression (Wichmann et al., 1996; Wunderlich et al., 2002). Testosterone is primarily synthesised in the gonads and increases susceptibility to various bacterial, viral, and parasitic diseases (Bernin and Lotter, 2014; Kadel and Kovats, 2018), including malaria (Benten et al., 1997). When testosterone interacts with its receptor on different immune response cells, it modifies the expression of genes that modulate cell proliferation, differentiation, and cytokine concentration (Ahmed et al., 1987; Bianchi, 2019). This steroid affects the development and number of T-cell subpopulations (Roden et al., 2004). It increases CD8^+,^ decreases CD4^+,^ and inhibits B-cell maturation (Olsen and Kovacs, 2001). Testosterone also reduces NK cell activity in both sexes (Hou and Zheng, 1988). The effects of testosterone depend on its concentration, which changes with age and sex (Gubbels Bupp et al., 2018). Generally, androgens upregulate the expression of Th2-type cytokines in male mice, while in females, they promote the increase of Th1-type cytokines (Bianchi, 2019). In malaria, testosterone administration increases mortality in C57/Bl10 mice infected with *Plasmodium chabaudi* and IgG antibody synthesis and decreases the population of CD8^+^ lymphocyte (Benten et al., 1991; Legorreta-Herrera et al., 2015). Moreover, gonadectomy enhances survival in male mice infected with *Plasmodium berghei* ANKA (Legorreta-Herrera et al., 2015). However, most of these experiments were carried out on one sex only; it is noteworthy that, even though sexual dimorphism in malaria has been recognised (Wunderlich et al., 1991; Wunderlich et al., 1993; Landgraf et al., 1994), in general, sex is not considered a variable and most studies are conducted on a single-sex. Therefore, very little is known about the contribution of testosterone to the sexual dimorphism of the immune response in malaria. Hence, we examined whether the increase in its concentration by exogenous administration or the decrease in testosterone concentration by gonadectomy prior to *P. berghei* ANKA infection affects parasitaemia, haemoglobin levels, body weight, and body temperature in male and female mice infected with the parasite. In addition, to analyse the effects of this hormone on the immune response, we quantified the subtypes of T lymphocytes, B lymphocytes, macrophages, and NK cells in the spleen; we included the analysis of the plasma concentration of the cytokines IFN-γ, TNF-α, IL-6, IL-10 and the levels of IgM and IgG antibodies. The findings of this study allow us to learn more about the role of testosterone in the sexual dimorphism that occurs in both pathology and immune response in malaria.

## Experimental design

To assess the contribution of testosterone to sexual dimorphism in the immune response to malaria, we developed a cross-sectional and comparative study of three strategies. First, we increased the testosterone levels through exogenous administration in intact male and female mice. Second, in other groups of male and female mice, we removed the gonads to generate testosterone-deficient mice. All gonadectomized mice were used after four weeks to help them recover from surgery. Third, groups of gonadectomised (GX) male and female mice were reconstituted with testosterone four weeks after surgery. We included intact or GX mice treated with vehicle (sweet almond oil) as controls. Each group consisted of 10 CBA/Ca 12-weeks-old female or male mice. On the day after the last administration of testosterone or vehicle (control groups), mice in each group were separated into two subgroups, one was infected with 1×10^3^ erythrocytes parasitized with *Plasmodium berghei* ANKA (*P. berghei* ANKA). Finally, the other subgroup was injected with PBS as infection control group. The whole experiment was performed twice (n = 10). Because the *P. berghei* ANKA infection is lethal, and all mice die between day 9 and 11 post-infection; we sacrificed them on day eight post-infection.

## Materials and methods

### Mice

CBA/Ca mice were a generous gift from Dr William Jarra (National Institute for Medical Research, London, UK). Mice were kept, fed, raised, and maintained in an environment free of specific pathogens in the animal facilities of the FES Zaragoza, UNAM. The protocol received approbation by the Local Ethics Committee (registration number 28/04/SO/3.4.1) based on the Mexican official standard NOM-062-ZOO-1999 for the use and care of laboratory animals. Euthanasia of the experimental animals was conducted humanely through cervical dislocation following anaesthesia with 5% sevoflurane (Abbot, Ciudad de México, Mexico).

### Parasite and infection

*Plasmodium berghei* ANKA was a kind donation from Dr William Jarra and stored in liquid nitrogen. To reactivate the parasite, a vial was thawed and immediately intraperitoneally injected into a CBA/Ca mouse. When the parasitaemia reached 20%, blood was drawn and diluted with Krebs’ solution. For infection, 100 µL containing 1×10^3^ parasitized erythrocytes were intravenously injected per mouse.

### Ovariectomy

The procedures were performed in accordance with the above (Legorreta-Herrera et al., 2015). In summary, four-week-old female mice were sedated with ketamine (100 mg/Kg, Vetoquinol, Cedex, France) and xylazine (15 mg/Kg, Pisa, Guadalajara, México). The ovaries were removed, and the abdomen was stitched. Mice were set aside to recover from surgery over four weeks. The lack of ovaries was confirmed by visual examination. The entire experiment was replicated twice with 5 female mice per group in each experiment (n = 10).

### Orchiectomy

Four-week-old male mice were given ketamine (100 mg/Kg, Vetoquinol, Cedex, France) and xylazine (15 mg/Kg, Pisa, Guadalajara, México), and their testes were removed through scrotal incisions as described above (Legorreta-Herrera et al., 2015). The efferent ducts were electrocauterized, and the testicles and epididymis were detached in sterile conditions. The mice were used after four weeks to help mice recover after surgery. The entire design was performed twice with 5 male mice per group in each trial (n = 10).

### Testosterone treatment

Each mouse was administered with 0.9 mg of testosterone (Schering Plough, Newton, NJ, US) or vehicle [sweet almond oil (LASA, Ciudad de México, Mexico)] subcutaneously every 72 h for 3 weeks according to Benten et al. (1991). The day after the last testosterone treatment, mice were infected with 1×10^3^ *P. berghei* ANKA parasitized erythrocytes.

### Parasitaemia

The parasite load was assessed using thin blood smears fixed with absolute methanol and stained with Giemsa (Merck, Darmstadt, Germany). Parasitized red blood cells were observed on a Zeiss Standard microscope (Carl Zeiss Ltd, Hertfordshire, Welwyn Garden City, UK) using the 100 X oil immersion lens. The parasitaemia results show the geometric mean of the number of parasitized red blood cells in 50 fields ± SEM. The entire experiment was conducted twice (n = 10) for each group.

### Body temperature

Body temperature was assessed from day 0 to day 8 post-infection using an infrared thermometer (Thermofocus, 01500A/H1N1, Vedano Olana-Varese, Italy). The thermometer beam was directed 5 cm from the ventral region of the mouse. Each point represents the mean ± SEM in each group. The whole experiment was performed twice (n = 10).

### Weight change

Weight was evaluated daily using a semi-analytical scale (Ohaus Pine Brook, NJ, US); the body weight detected on day zero post-infection was considered the 100%. The weight change was calculated using the mouse weight on each day post-infection compared with the weight on day 0 using the day the following equation:


$$Change\;\left(percentage\right)=\;\frac{\left(Wx-W0\right)\times 100}{W0}$$

Where:

*Wx* is the weight on the analysis day, and *W*0 is the weight on day 0 post-infection.

### Haemoglobin concentration

Two microlitres of tail mouse blood were mixed with 498 μL of Drabkin reagent [0.77 mM potassium cyanide (KCN) (Sigma-Aldrich, St Louis, MO, US), 0.61 mM potassium ferricyanide K_3_Fe(CN)_6_ (Sigma-Aldrich), and 12 mM sodium bicarbonate (NaHCO_3_) (Sigma-Aldrich)], mixed vigorously, and incubated protected from light for 5 minutes at room temperature. The haemoglobin optical density was measured by spectrophotometry at 540 nm; a standard curve based on a commercial haemoglobin standard (Sigma-Aldrich) was used to calculate the haemoglobin concentration.

### Quantification of spleen cell populations using flow cytometry

Groups of intact or gonadectomized (GX) mice of both sexes were treated with vehicle or testosterone for three weeks. The day after the last testosterone treatment, mice were allocated into two subgroups. The first group was infected with 1×10^3^ *P. berghei* ANKA parasitized erythrocytes. The second group was uninfected to assess the effects of testosterone in the absence of infection. Mice were sacrificed eight days later, and their spleen was dissected and disaggregated using a nylon mesh. The spleen cells were washed with 500 μL of cold PBS and fixed for 10 minutes using the commercial solution (Becton and Dickinson, Franklin Lakes, NJ, USA). The cells were washed with staining solution (PBS, 1% albumin, 0.1% NaN_3_) and counted in a Neubauer chamber. 1×10^6^ cells were stained in the dark for 30 minutes using the following dilutions of anti-mouse fluorochrome-coupled antibodies: 1:250 for FITC-antiCD3; 1:1000 for APC-antiCD4; 1:1000 for PE-antiCD8; 1:1600 for APC-anti B220; 1:125 for PE-AntiMac3, and 1:160 for PE anti CD16^+^/32^+^. For this purpose, three mixtures of antibodies were prepared: the first identified total T cells (CD3^+^, CD4^+^, and CD8^+^), the second mixture identified B cells and macrophages (B220^+^ and anti-Mac3^+^), and the third (B220^-^CD3^-^CD16^+^/32^+^) to identify NK cells. Finally, the cells were washed with PBS and resuspended on 100 μL of FACS solution to be acquired on the flow cytometer FACS Aria II (BD Biosciences, San Jose, California, USA). Three tubes were used for each population; the control of unstained cells, the control of cells stained with a single antibody coupled to the fluorochrome and the tube containing the isotype control.

For immunotyping, we stained spleen cells with three different antibody mixtures. In the first mixture, we identified total T cells (CD3^+^), helper T cells (CD4^+^) and cytotoxic T cells (CD8^+^). For this purpose, we acquired 10 000 events per sample, then plotted FSSC vs SSC and selected the uniform region corresponding to lymphocytes, thus eliminating doublets and singlets; this first gate was equivalent to 100%. From this region, we selected FITC-CD3^+^ (positive lymphocytes) by plotting SSC vs FITC-CD3^+^ and calculated the percentage relative to the first gate (this procedure excludes B lymphocytes and NK cells) (A). From the first gate, two more dot plots were made, corresponding to CD4^+^ and CD8^+^ lymphocytes (SSC vs APC-CD4^+^ and SSC vs PE-CD8^+^ respectively) and the percentages were calculated in relation to the initial lymphocytes’ gate, the sum of the percentages of CD4^+^ and CD8^+^ cells correspond to the total CD3^+^ lymphocytes.

In the second mixture, B cells (B220^+^) and macrophages (Mac3^+^) were identified with APC-antiB220 and PE-antiMac3 antibodies, respectively. The B220^+^ population was selected from the lymphocyte region using FSS vs SSC dot plot and corresponded to 100%. Subsequently, with the SSC vs APC-B220 dot plot, B220^+^ cells were selected. Monocytes were selected based on FSC and SSC which corresponds to a population above lymphocytes and this region was 100%. The latter procedure excluded CD3^+^, B220^+^ and NK cells, from which region macrophages were selected with a dot blot of SSC vs PE-antiMac3.

Finally, the third mixture was used to identify NK cells. For this purpose, we performed four dot blots: In the first we selected the lymphocyte and NK cell population by plotting SSC vs FSC; in the second dot blot we used the FITC-antiCD3 antibody to select the CD3^-^ cell population, which is where the NK cells are located; in the third dot blot we used the APC-Anti B220 antibody and selected the B220 negative cells. Finally, in the fourth dot blot, we plotted SSC against PE-CD16^+^/32^+^. All antibodies were purchased from Biolegend (Biolegend, San Diego, CA, USA). Stained cells were analysed using a FACSAria II flow cytometer (Beckton and Dickinson, San Jose, CA, USA); the data were examined with the FlowJo™ software (Beckton and Dickinson, Ashland, OR, USA).

### Th1/Th2/Th17 cytokine quantification

The levels of cytokines IL-2, IL-4, IL-6, IL-10, TNF-α, IFN-γ, and IL-17α were quantified using the commercial bead array method (BD Mouse Th1/Th2/Th17 CBA Kit Biosciences-Pharmingen, Heidelberg, Germany). Briefly, 50 μL of plasma were incubated in the dark with 50 μL of the bead mix and the detection reagent for 2 hrs at room temperature protected from light. The mixture was centrifuged at 2800 g for 5 minutes, and the supernatant was removed. The beads were washed in FACS solution and resuspended in 100 µL of FACS liquid. The concentration of each cytokine was calculated using a commercial standard curve.

### Extraction of plasma samples for quantification of sex steroids concentration

On day eight post-infection, mice were sacrificed, and blood was collected in tubes containing heparin. Samples were centrifuged at 1000 g for 5 minutes and the plasma was separated, aliquots were prepared to quantify free testosterone, dehydroepiandrosterone (DHEA) and 17β-oestradiol. 100 μL of plasma were vigorously mixed with 5 mL of ethyl ether (JT Baker, Fisher Scientific SL, USA) for 5 minutes. The aqueous phase was frozen in a dry ice bath with absolute ethanol (Sigma Aldrich), and the organic phase was transferred to a glass tube, the ether was evaporated into a 37°C water bath for 48 hours. Samples were rehydrated with 1000 µL of PBS/0.1% gelatine. With this dilution, the sample concentrations are within the range of the quantification methods.

### Quantification of testosterone

Free testosterone was quantified with the commercial reagents: free testosterone EIA-2924 with a sensitivity of 0.04 pg (DRG International, Frauenbergstr, Germany). 20 µL of the steroid extract or curve standards with 100 µL of conjugate were added to each well of the plate and incubated for 1 hour at 37°C. Afterwards, the plate was thoroughly washed with washing solution. 100 µL of tetramethylbenzidine (TMB) were added as substrate and incubated at 28°C in the dark for 15 minutes. To halt the reaction, 100 µL of stop solution was added, and after 5 minutes, the plate absorbance was read at 620 nm on the Multiskan Ascent 96 plate reader (Thermo Fisher Scientific, Waltham, MA, US). Testosterone concentrations were calculated using a standard curve included in each kit.

### Quantification of DHEA

DHEA hormone was quantified with the commercial DHEA kit EIA-3415 (Detection range: 0.07-30 ng/mL, sensitivity: 0.07 ng), (DRG International). 10 µL of extract or 10 µL of standard with 100 µL of conjugate per well were added to the plate. It was incubated for 1 hour at room temperature. Subsequently, the plate was washed four times with 400 µL of washing solution. 100 µl TMB was added as substrate and incubated at 28°C for 15 minutes. The reaction was halted with 100 µl stop solution and after 5 minutes the plate was read at 620-630 nm. Plate absorbance was read on the Multiskan Ascent 96 plate reader (Thermo Fisher Scientific). DHEA levels were calculated using a standard curve included in the kit.

### Quantification of 17β-oestradiol

A Siemens Immulite LKE2 solid phase competitive chemiluminescent immunoassay for oestradiol (Detection range: 20-2000 pg/mL, sensitivity: 15 pg) (Immulite 1000 Siemens Llanberis Gwynedd, UK) was used to quantify 17β-oestradiol. 500 µL of the sample extract was added to the reaction cuvettes and processed by the automated equipment. The results were quantified on the Immulite 1000 Immunoassay System (Siemens Healthineers, Surrey, UK).

### Statistical analysis

Repeated mean ANOVA with a Bonferroni *post-hoc* test was used for parasitaemia, weight loss, temperature, and haemoglobin concentration. Cell populations: CD3^+^, CD4^+^, CD8^+^, B220^+^, B220^-^CD3^-^CD16/32^+^ and Mac3^+^ in the spleen; IFN-γ, TNF-α, IL-6, IL-10, testosterone, DHEA, and 17β-oestradiol levels were analysed using the Kruskal-Wallis test with a Dunn *post-hoc*, using the SPSS 21 (IBM, Santa Fe, USA) and GraphPad Prism 9 programs (GraphPad Software, San Diego, California, USA).

## Results

### Testosterone administration generated dimorphic patterns in free testosterone and DHEA concentrations in intact *P. berghei* ANKA-infected mice

Testosterone is produced in females and males; it is transformed by 5α-reductase to dihydrotestosterone and by aromatase to 17β-oestradiol in gonads (Hammes and Levin, 2019). To find out whether exogenous administration of testosterone impacts the concentration of DHEA (testosterone precursor hormone), or whether its administration promotes its conversion to 17β-oestradiol, we used the three strategies mentioned above. We first quantified the concentration of free testosterone (biologically active fraction) (Sakiyama et al., 1988), 17β-oestradiol and the concentration of the precursor hormone dehydroepiandrosterone (DHEA) in all groups of mice.

**Figure 1:**
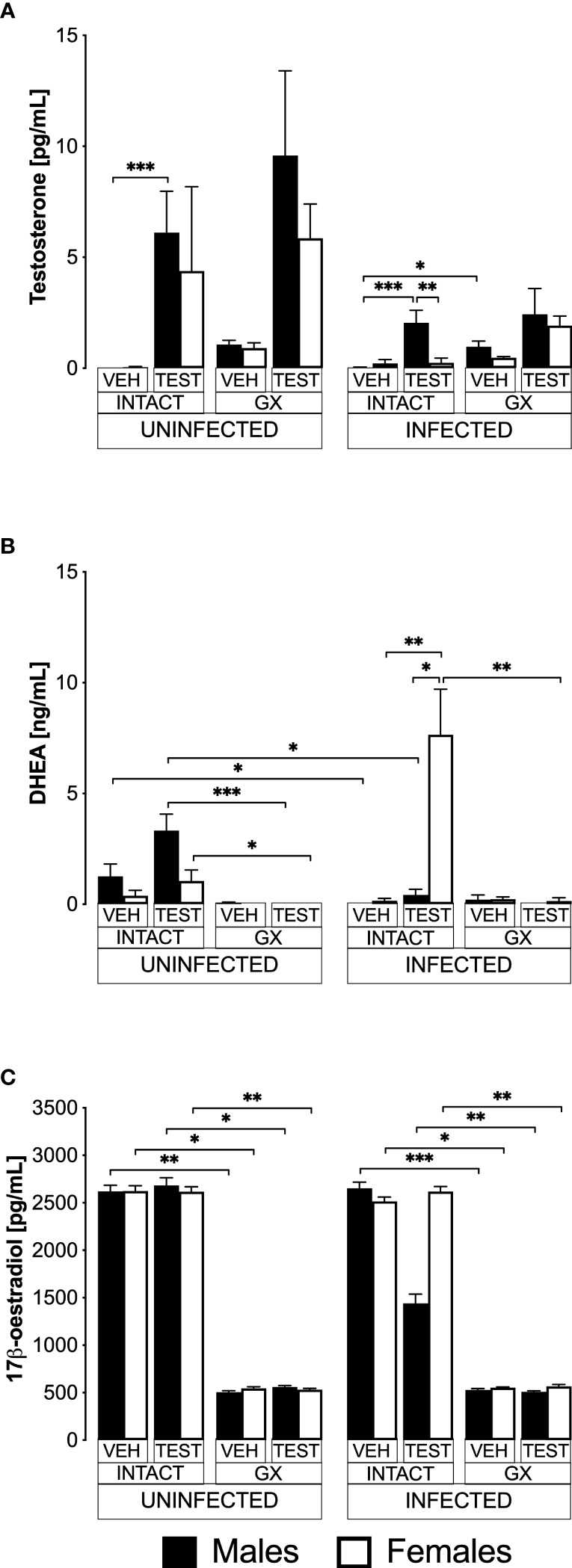
Testosterone generated a dimorphic pattern of free testosterone and dehydroepiandrosterone (DHEA) levels in intact CBA/Ca mice infected with *Plasmodium berghei* ANKA. Eight groups of males and eight groups of females were analysed. Four groups of each sex were gonadectomised (GX), and the other four groups were left intact. Four weeks later, two intact and two GX groups of each sex received testosterone for three weeks, and the other two groups received vehicle. In addition, one intact and one GX group treated with testosterone and one intact and one GX group treated with the vehicle of each sex were infected with *P. berghei* ANKA. Finally, the remaining groups were injected with PBS for infection control. All mice were sacrificed on day eight post-infection, blood was collected in heparinised tubes, centrifuged, and plasma was frozen until use. Sex steroids were extracted from the plasma with ethyl ether and hydrated with PBS/gelatine. Free testosterone concentration (sensitivity: 0.04 pg) is shown in **(A)**, DHEA concentration (sensitivity: 0.07 ng) in **(B)**, and 17β-oestradiol concentration (sensitivity: 15 pg) in **(C)**. Each bar represents the mean ± SEM of the following groups: the vehicle-treated (VEH), the testosterone-treated (TEST), the intact (INTACT), or the gonadectomised (GX). Mice infected with *P. berghei* ANKA (INFECTED) or uninfected (UNINFECTED). Asterisks represent statistically significant differences between groups (*) p<0.05; (**) p<0.01; (***) p<0.001; calculated by Kruskal-Wallis analysis with Dunn’s *post-hoc* test. The whole experiment was performed twice (n = 10).

Administering testosterone increased free testosterone levels in uninfected intact males, without affecting females in the same condition (**Figure 1A**). However, gonadectomy or reconstituting GX mice with testosterone did not change the testosterone concentration in both sexes. Administering testosterone increased the concentration of free testosterone in intact infected males without affecting females in the same condition, resulting in a dimorphic pattern. Finally, the GX group treated with the vehicle and infected increased the concentration of free testosterone compared to the intact male group treated with vehicle and infected (**Figure 1A**).

Regarding DHEA, in the uninfected intact mice, administering testosterone did not change DHEA concentration (**Figure 1B**). Gonadectomy reduced DHEA concentration in uninfected mice treated with testosterone of both sexes, and reconstituting GX mice with testosterone did not restore their DHEA concentration. In contrast, the group of intact female mice treated with testosterone and infected with *P. berghei* ANKA significantly increased their DHEA concentration, resulting in a dimorphic pattern. In addition, in the group of GX females treated with testosterone and infected, the concentration of DHEA decreased (**Figure 1B**).

Regarding 17β-oestradiol concentration, administration of testosterone to uninfected intact mice did not change the levels of this hormone in either sex. Furthermore, gonadectomy decreased 17β-oestradiol concentration in both sexes and treating GX mice with testosterone did not restore their 17β-oestradiol levels. Finally, administering testosterone to either intact or GX infected mice did not change their 17β-oestradiol levels (**Figure 1C**).

### Testosterone generated a dimorphic pattern on parasitaemia of intact female mice and reduced parasitaemia in gonadectomised CBA/Ca female mice infected with *P. berghei* ANKA

**Figure 2:**
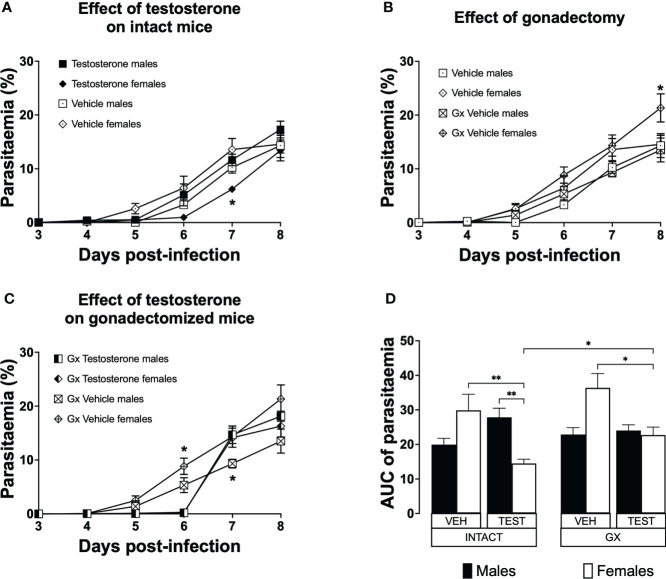
Testosterone decreased parasitaemia in intact or gonadectomised female mice infected with *P. berghei* ANKA. Four groups of males and four groups of females were used. Two groups of each sex were gonadectomised (GX), and the other two groups were left intact. Four weeks later, one intact and one GX group of each sex received testosterone for three weeks, and the other intact or GX group received vehicle. Finally, all groups were infected with *P. berghei* ANKA. Parasitaemia was assessed in Giemsa-stained blood smears from day 3 to 8 post-infection. **(A)** shows the effect of administering testosterone to intact mice; **(B)** represents the effect of gonadectomy in vehicle-treated mice; **(C)** shows the effect of reconstituting GX mice with testosterone. Each point represents the mean parasitaemia in each group ± SEM on days 3 to 8 post-infection. Asterisks represent statistically significant differences obtained by repeated measures ANOVA with a Bonferroni *post-hoc* test (*) (n = 10) p ≤ 0.05; **(D)** shows the result of the area under the curve (AUC) results of the vehicle (VEH) or testosterone (TEST), intact (INTACT) or gonadectomised (GX) groups. Each bar represents the geometric mean ± SEM. Asterisks represent significant difference between groups: (*) p < 0.05; (**) p<0.01; calculated by Kruskal-Wallis analysis with Dunn *post-hoc* test. The whole experiment was performed twice (n = 10).

Parasitaemia is a measure of parasite proliferation in the blood (Giemsa, 1902). To study the effect of testosterone on parasitaemia, we used the three strategies mentioned before. Parasitaemia was plotted from day 3 to day eight post-infection. To summarize the effect of treatments during the whole infection, the area under the curve for each group was calculated and plotted as a histogram. Intact females treated with vehicle developed parasitaemia like that of males in the same condition (**Figure 2A**). However, intact females treated with testosterone delayed the initial increase in parasitaemia and had lower parasitaemia compared to intact males receiving testosterone on day 7 post-infection (**Figure 2A**). Gonadectomy did not modify parasitaemia in male mice; in contrast, it increased parasitaemia in females on day 8 post-infection (**Figure 2B**). In addition, vehicle-treated GX females had higher parasitaemia than testosterone-treated GX females on day 6 post infection (**Figure 2C**). Remarkably, reconstituting GX animals with testosterone delayed the initial increase in parasitaemia in both sexes until day 6 post infection (**Figure 2C**). Finally, reconstituting GX females with testosterone reduced parasitaemia in females only, eliminating the dimorphic pattern generated by gonadectomy (**Figure 2D**).

### Testosterone reduced haemoglobin concentration in *P. berghei* ANKA-infected male mice

**Figure 3:**
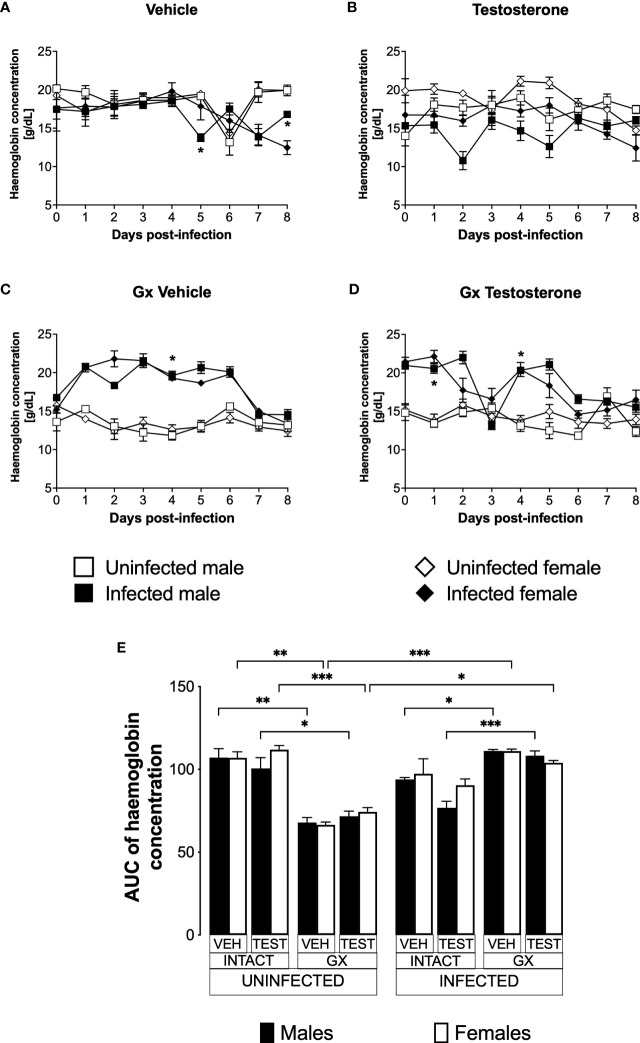
Gonadectomy prevented haemoglobin level reduction in mice infected with *P. berghei* ANKA. We used 8 groups of males and 8 groups of females, 4 groups of each sex were gonadectomised (GX), and the other 4 groups were left intact; four weeks after surgery, two groups of intact and two GX mice of each sex were administered testosterone for 3 weeks, the other two received vehicle for the same time. Finally, one intact and one GX group treated with testosterone of each sex were infected with *P. berghei* ANKA. The remaining groups were injected with PBS as negative infection controls. From the day of infection, haemoglobin concentration was quantified in all groups. **(A)** shows haemoglobin concentration in vehicle-treated male and female intact mice infected or uninfected with the parasite; **(B)** shows haemoglobin concentration in testosterone-treated male and female intact mice groups; **(C)** shows haemoglobin concentration in vehicle-treated GX groups infected or uninfected in both sexes; **(D)** shows haemoglobin concentration in the testosterone-treated GX groups infected or uninfected. Asterisks represent statistically significant differences between uninfected and infected groups, obtained by repeated means ANOVA with a Bonferroni *post-hoc* test (*) p ≤ 0.05. Finally, **(E)** shows the area under the curve analysis (AUC) of the vehicle-treated (VEH), testosterone-treated (TEST), intact (INTACT), or gonadectomised (GX) groups. Mice infected with *P. berghei* ANKA (INFECTED) or uninfected (UNINFECTED). Each bar represents the mean ± SEM. Asterisks denote statistical significance between 2 groups (*) p<0.05; (**) p<0.01; (***) p<0.001; calculated by Kruskal-Wallis analysis with Dunn’s *post-hoc* test. The whole experiment was performed twice (n=10).

Anaemic individuals have low haemoglobin levels, a condition that is characteristic of malaria (Maggio-Price et al., 1985). Given that testosterone promotes erythropoiesis in a dose-dependent manner (Coviello et al., 2008); in this work, we evaluated the effect of testosterone on haemoglobin concentration which is an indirect form of anaemia detection. We first assessed the impact of testosterone on haemoglobin concentration in uninfected intact or GX male and female mice. We detected no difference in haemoglobin concentration between intact males and females treated with vehicle (**Figure 3A**). Administering testosterone to uninfected intact mice did not change haemoglobin concentration (**Figure 3B**). In contrast, gonadectomy decreased haemoglobin concentration in uninfected males and females (**Figure 3C, E**) and reconstituting GX mice with testosterone did not restore their initial haemoglobin concentration (**Figure 3D, E**). Next, we analysed the effect of testosterone on haemoglobin concentration in infected intact or GX mice. Intact males infected with *Plasmodium berghei* ANKA decreased haemoglobin concentration on day five post-infection compared to females in the same condition (**Figure 3A**). Testosterone administration to infected intact mice did not change haemoglobin concentration (**Figure 3B**). Contrary to our expectations, infected GX females and males increased haemoglobin concentration compared to uninfected GX females (**Figure 3C**). Finally, reconstitution of GX mice with testosterone did not modify haemoglobin concentration (**Figure 3D, E**).

### Testosterone increased body weight in intact female mice infected with *P. berghei* ANKA

**Figure 4:**
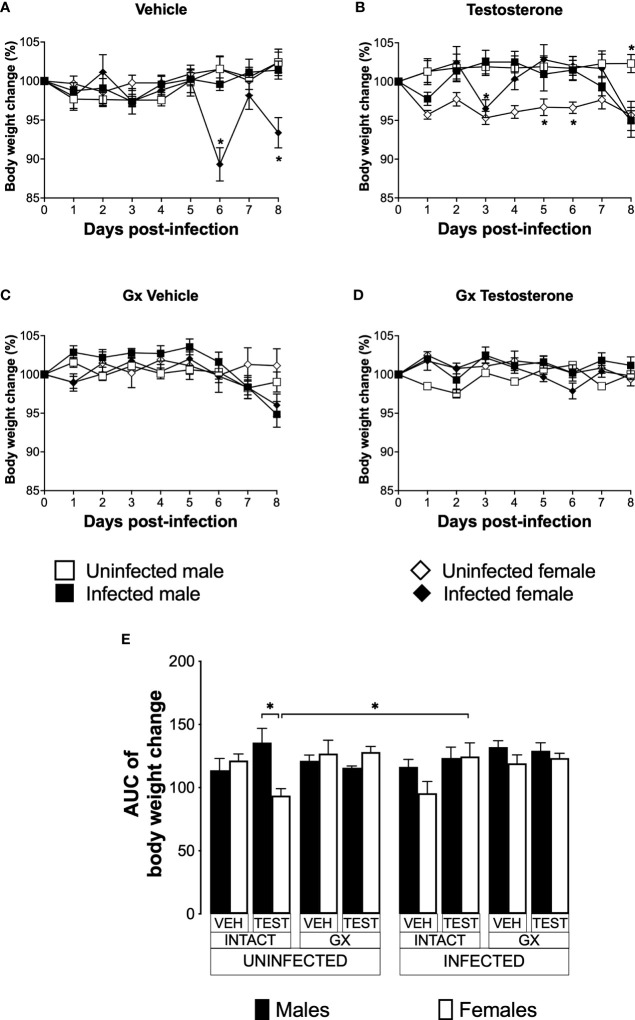
Testosterone increased body weight in female mice infected with *P. berghei* ANKA. We studied 8 groups of males and 8 groups of females, 4 groups of each sex were gonadectomised (GX), and the other 4 groups were left intact; four weeks after surgery, two groups of intact and two GX mice of each sex were administered testosterone for 3 weeks, the other two received vehicle for the same time. Finally, one intact and one GX group treated with testosterone of each sex were infected with *P. berghei* ANKA. The remaining groups were injected with PBS as negative infection controls. From the onset of infection, the percentage change in body weight was determined on the base of the body weight obtained at day zero of infection (100%). **(A)** shows the body weight change in intact male and female mice infected or uninfected with the parasite and treated with vehicle; **(B)** shows body weight change in testosterone-treated male and female intact mice groups; **(C)** shows body weight change in vehicle-treated GX groups infected or uninfected in both sexes; **(D)** shows body weight change in the testosterone-treated GX groups infected or uninfected. Asterisks (*) represent statistically significant differences between uninfected and infected groups, obtained by repeated means ANOVA with a Bonferroni *post-hoc* test p ≤ 0.05. Finally, **(E)** shows the area under the curve analysis (AUC) of the vehicle-treated (VEH), testosterone-treated (TEST), intact (INTACT), or gonadectomised (GX) groups. Mice infected with *P. berghei* ANKA (INFECTED) or uninfected (UNINFECTED). Each bar represents the mean ± SEM. Asterisks denote statistical significance between 2 groups (*) p < 0.05; calculated by Kruskal-Wallis analysis with Dunn’s *post-hoc* test. The whole experiment was conducted in duplicate (n = 10).

Weight loss is a feature of malaria (Basir et al., 2012); furthermore, testosterone reduces the body weight in mice (Sebo and Rodeheffer, 2021). To assess the effect of testosterone on body weight loss, we used the three strategies mentioned before. The weight of uninfected intact males and females did not change over the nine days analysed (**Figure 4A**). Testosterone administration to uninfected intact mice reduced the body weight in females on days 5 and 6 without affecting males, resulting in a dimorphic pattern (**Figure 4B, E**). Gonadectomy did not affect the body weight in either group (**Figure 4C**). Finally, testosterone reconstitution of GX mice did not alter body weight in either sex (**Figure 4D, E**).

Infected intact females reduced body weight on days 6 and 8 post-infection compared to uninfected intact females. In contrast, infected intact males did not change their body weight (**Figure 4A**); interestingly, the administration of testosterone to infected females prevented them from reducing their body weight. In contrast, testosterone-treated infected intact males decreased their body weight on day eight post-infection compared to uninfected intact mice (**Figure 4B**). Gonadectomy did not change body weight in infected mice (**Figure 4C**). Finally, reconstituting infected GX mice with testosterone did not alter body weight in either sex (**Figure 4D, E**).

### Infection increased the temperature of male and female GX mice

Increased body temperature is characteristic of human malaria, mainly associated to the release of proinflammatory cytokines with the activity of endogenous pyrogens (Ejezie and Ezedinachi, 1992). However, murine models of malaria are characterized by the development of severe hypothermia (Curfs et al., 1993; Cernetich et al., 2006). To evaluate the effect of testosterone on temperature, we used the three strategies mentioned before.

**Figure 5:**
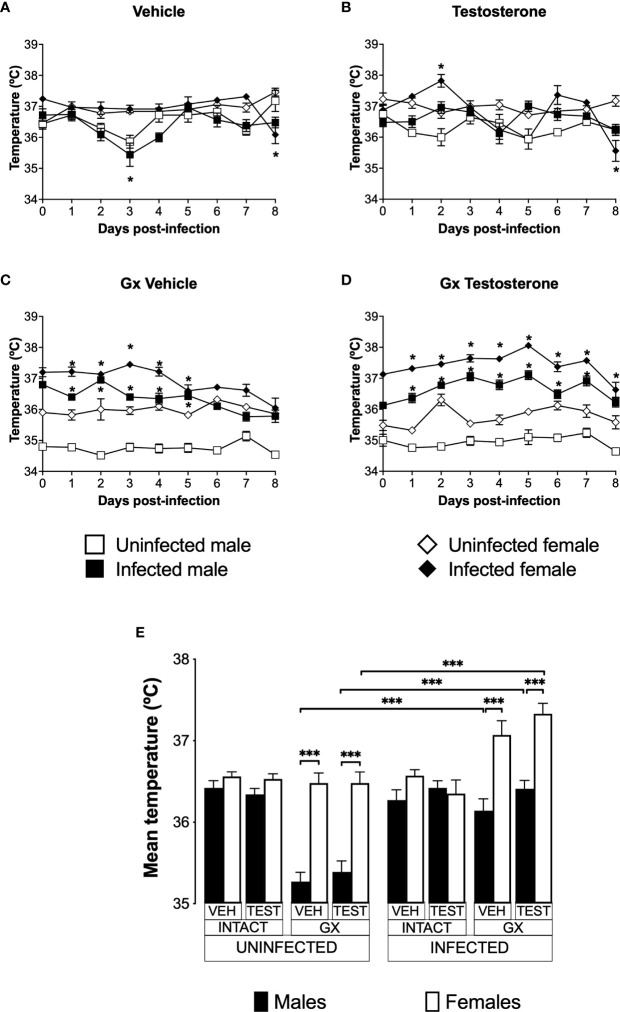
Gonadectomy generated a dimorphic pattern in the temperature of mice infected with *P. berghei* ANKA. We analysed 8 groups of males and 8 groups of females, 4 groups of each sex were gonadectomised (GX), and the other 4 groups were left intact; four weeks after surgery, two groups of intact and two GX mice of each sex were administered testosterone for 3 weeks, the other two received vehicle for the same time. Finally, one intact and one GX group treated with testosterone of each sex were infected with *P. berghei* ANKA. The remaining groups were injected with PBS as infection controls. Temperature was measured in all groups from the day cero to day eight post-infection. **(A)** shows the temperature in intact male and female mice treated with vehicle that were infected or uninfected with the parasite; **(B)** displays the temperature in groups of male and female intact mice treated with testosterone infected or no with *P. berghei* ANKA; **(C)** shows the temperature in GX mice treated with vehicle infected or uninfected of both sexes; **(D)** indicates the temperature in the GX mice treated with testosterone infected or not with the parasite. Each point on the graphs represents the average ± SEM in each group. Asterisks (*) represent statistically significant differences between uninfected and infected groups, obtained by repeated measures ANOVA with a Bonferroni *post-hoc* test p ≤ 0.05. Finally, **(E)** shows the area under the curve analysis (AUC) of the vehicle-treated (VEH), testosterone-treated (TEST), intact (INTACT), or gonadectomised (GX) groups. Mice infected with *P. berghei* ANKA (INFECTED) or uninfected (UNINFECTED). Each bar represents the mean ± SEM. Asterisks denote statistical significance between 2 groups (*) p < 0.05; (***) p < 0.001; calculated by Kruskal-Wallis analysis with Dunn’s *post-hoc* test. The whole experiment was conducted in duplicate (n = 10).

Uninfected and vehicle-treated intact females did not change the temperature from day 0 to day 8 (**Figure 5A**). Testosterone administration did not affect the temperature in either infected or uninfected intact males. However, intact testosterone-treated and infected females increased in temperature on day 2 post-infection (**Figure 5B**). In contrast, gonadectomy significantly reduced temperature in male mice uninfected treated with vehicle or with testosterone (**Figure 5C**), resulting in a marked dimorphic pattern (**Figure 5C, E**). Finally, reconstitution of GX mice with testosterone did not change the temperature in uninfected mice, preserving the dimorphic pattern (**Figure 5D, E**).

Infection did not change temperature in intact mice of either sex except by day 8 in females (**Figure 5A**). However, intact testosterone-treated and infected females exhibited significant variations in body temperature; on day 2 post-infection they increased in temperature (**Figure 5B**); nevertheless, on day 8 they decreased in temperature compared to uninfected intact females. In contrast, testosterone did not modify temperature in the infected intact male group (**Figure 5B**). In GX mice treated with vehicle or testosterone, infection increased temperature in both sexes (**Figure 5C, D**) resulting in a dimorphic pattern (**Figure 5E**).

### Testosterone affected CD3^+^, CD4^+^ and CD8^+^ T cell populations to a greater extent in females than in males

**Figure 6:**
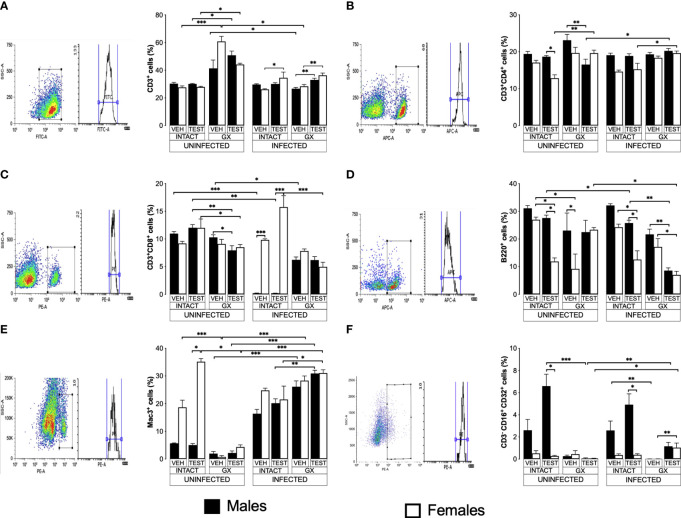
Testosterone increased CD3^+^, CD8^+^, B220^+^ and Mac3^+^ populations in females, whereas it increased only the NK in males. We used 8 groups of males and 8 groups of females, 4 groups of each sex were gonadectomised (GX), and the other 4 groups were left intact; four weeks after surgery, two groups of intact and two GX mice of each sex were administered testosterone for 3 weeks, the other two received vehicle for the same time. Finally, one intact and one GX group, both treated with testosterone of each sex were infected with *P. berghei* ANKA. The remaining groups were injected with PBS to be used as infection controls. Mice were sacrificed on day 8 post infection; spleens were removed and were used to quantify immune response cells by flow cytometry using population-specific monoclonal antibodies coupled to different fluorochromes. The dot plot shows the selection strategy of positive cells and the histogram for each population. In addition, the graph of the mean of the percentage of each cell population ± SEM for each group is presented. **(A)** shows total CD3^+^ T lymphocytes; **(B)** represents CD3^+^CD4^+^ helper T lymphocytes; **(C)** shows CD3^+^CD8^+^ cytotoxic T lymphocytes; **(D)** shows B220^+^ B lymphocytes; **(E)** represents Mac3^+^ macrophages, and **(F)** shows CD3^-^CD19^-^CD16^+^CD32^+^ (NK cells) of the vehicle-treated (VEH), testosterone-treated (TEST), intact (INTACT), or gonadectomised (GX) groups. Mice infected with *P. berghei* ANKA (INFECTED) or uninfected (UNINFECTED). Asterisks represent statistical significance between groups (*) p < 0.05; (**) p < 0.01; (***) p < 0.001; were calculated with a Kruskal-Wallis test with Dunn’s *post-hoc* test. The whole experiment was conducted in duplicate (n = 10).

To evaluate the effect of testosterone on immune response cells in the spleen, we used the same three strategies. We evaluated the effect of testosterone on the CD3^+^, CD3^+^CD4^+^, CD3^+^CD8^+^, B220^+^, Mac^+^ y NK cells subpopulations in the spleen because it is the organ where immune cells eliminate *Plasmodium* as reviewed by Ghosh (Ghosh and Stumhofer, 2021). In addition, testosterone decreases the maturation of Th1 cells *in vitro* (Massa et al., 2017). Testosterone administration was not found to affect the CD3^+^ population in uninfected mice; interestingly, gonadectomy significantly increased this population in uninfected females compared to intact uninfected males. However, reconstituting uninfected GX mice of both sexes with testosterone did not restore the CD3^+^ population to its basal levels (**Figure 6A**). Infection modified this pattern, and the administration of testosterone increased the CD3^+^ population only in intact infected females. In addition, GX mice of both sexes reconstituted with testosterone increased their CD3^+^ population compared with mice in the same condition treated with vehicle (**Figure 6A**).

Regarding CD3^+^CD4^+^ cells, intact testosterone-treated females uninfected had a lower number of CD3^+^CD4^+^ than intact males in the same condition, resulting in a dimorphic pattern (**Figure 6B**). In addition, gonadectomy did not modify the CD3^+^CD4^+^ population in uninfected mice, and the reconstitution with testosterone of GX males decreased the levels of CD3^+^CD4^+^ cells without affecting this cell population in female mice in the same condition (**Figure 6B**). In contrast, infection with *P. berghei* ANKA did not modify this population in intact or GX mice. (**Figure 6B**); However, the group of GX females reconstituted with testosterone and infected increased this population compared to intact females treated with testosterone (**Figure 6B**).

On the other hand, testosterone administration or gonadectomy did not affect the CD3^+^CD8^+^ population in the uninfected groups; however, reconstituting GX males with testosterone decreased this population (**Figure 6C**). Interestingly, infection produced a marked dimorphic pattern in this population; intact males had an extremely low numbers of these cells, while females in the same condition were unaffected (**Figure 6C**). In addition, intact testosterone-treated and infected females further increased this population, preserving the dimorphic pattern (**Figure 6C**). Finally, in the testosterone-treated and infected group of GX females, the CD3^+^CD8^+^ population decreased, which eliminated the dimorphic pattern (**Figure 6C**).

### Testosterone administration generated a dimorphic pattern in the B220^+^, Mac3^+^ and B220^-^CD3^-^CD16^+^CD32^+^ cells

B220^+^ cells are essential because they are precursors of cells that produce antibodies which contribute to the elimination of *Plasmodium* (White et al., 1991; Joos et al., 2010). Testosterone has been shown to decrease the migration of B cells from the bone marrow to the spleen and the number of antibody-producing cells *in vitro* and in the spleen (Kozlov et al., 1979; Sthoeger et al., 1988). Interestingly, in this work, testosterone administration to uninfected intact mice generated a dimorphic pattern in the B220^+^ population: intact females significantly decreased this cell population, while in intact males there was no change (**Figure 6D**). In addition, gonadectomy did not affect uninfected males but decreased this population in females resulting in a dimorphic pattern; furthermore, testosterone reconstitution of GX mice uninfected eliminated the dimorphic pattern (**Figure 6D**). Administration of testosterone to intact infected mice significantly reduced the number of B220^+^ cells, particularly in female mice generating a dimorphic pattern; however, gonadectomy did not affect this population. In addition, reconstitution of GX mice with testosterone decreased the levels of B220^+^ cells in both sexes (**Figure 6D**).

Additionally, we quantified the percentage of macrophages (Mac-3^+^) in the spleen because these cells are central for *Plasmodium* elimination through phagocytosis (Chua et al., 2013), and these cells possess testosterone receptors on their plasma membrane (Benten et al., 1999). Uninfected intact testosterone-treated females had higher numbers of macrophages than males in the same condition, resulting in a dimorphic pattern; however, gonadectomy decreased this population only in females and eliminated the dimorphic pattern. Reconstitution of GX mice with testosterone did not restore this cell population in females (**Figure 6E**). The infection increased the percentage of Mac-3^+^ cells in males (**Figure 6E**). The administration of testosterone to intact infected male or intact infected female mice did not change this population. However, testosterone-treated GX mice showed an increase compared to intact testosterone-treated and infected mice (**Figure 6E**).

On the other hand, we evaluated the percentage of NK cells in the spleen because this subpopulation of lymphocytes contributes to infection control through the release of proinflammatory cytokines and cytotoxic activity that destroys cells infected with *Plasmodium*, reviewed by Wolf et al. (2017). We detected that testosterone administration increased the NK cell population (B220^-^CD3^-^CD16^+^CD32^+^) in intact uninfected males, resulting in a dimorphic pattern, with males having a higher percentage of this population compared to females in the same condition. It is interestingly to note that gonadectomy only affected males by decreasing this population, eliminating the dimorphic pattern (**Figure 6F**). Administering testosterone to intact infected mice increased the NK cell population (B220^-^CD3^-^CD16^+^CD32^+^) only in males, resulting in a dimorphic pattern. Interestingly, gonadectomy only affected males in which this population was almost depleted, eliminating the dimorphic profile. Finally, NK cell percentage was not restored when GX males were reconstituted with testosterone; however, GX females treated with testosterone and infected with the parasite increased this population (**Figure 6F**).

### Testosterone decreased IFN-γ levels and increased IL-6 in infected intact females

**Figure 7:**
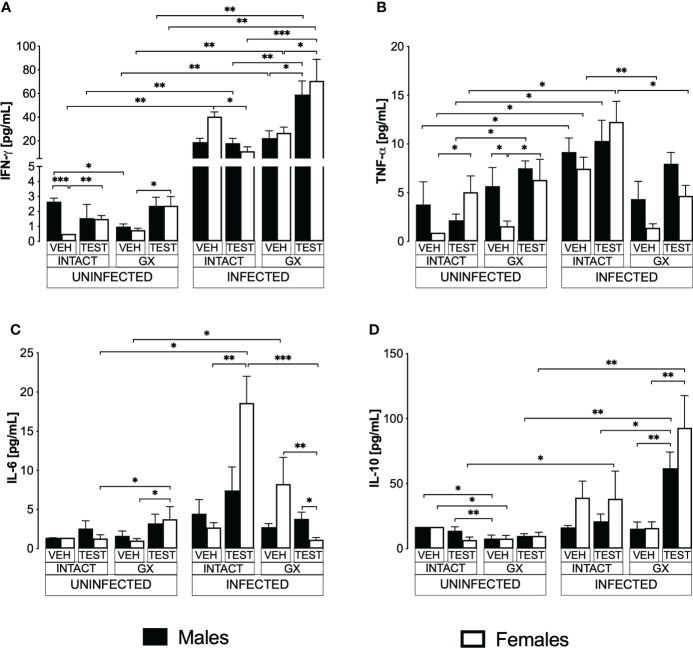
Modifying testosterone concentration affected IFN-γ, TNF-α, and IL-6 levels in *P. berghei* ANKA-infected females. We used 8 groups of males and 8 groups of females, 4 groups of each sex were gonadectomised (GX), and the other 4 groups were left intact; four weeks after surgery, two groups of intact and two GX mice of each sex were administered testosterone for 3 weeks, the other two received vehicle for the same time. Finally, one intact (INTACT) and one GX group treated with testosterone of each sex were infected with *P. berghei* ANKA. The remaining groups were injected with PBS as infection controls. On day 8 post-infection, mice were sacrificed, blood was removed, and plasma was used to assess the concentration of IFN-γ **(A)**, TNF-α **(B)**, IL-6 **(C)** and IL-10 **(D)** using flow cytometry. Histograms represent the mean ± SEM of each cytokine plasma level in the vehicle-treated (VEH), testosterone-treated (TEST), intact (INTACT), or gonadectomised (GX) groups. Mice infected with *P. berghei* ANKA (INFECTED) or uninfected (UNINFECTED). Asterisks represent statistical significance between groups (*) p < 0.05; (**) p < 0.01; (***) p < 0.001; were calculated with a Kruskal-Wallis test with Dunn’s *post-hoc* test. The whole experiment was conducted in duplicate (n = 10).

Pro- and anti-inflammatory cytokines are essential mediators in the immune response to *Plasmodium*; particularly, IFN-γ modulates disease progression by activating macrophages to eliminate parasites through phagocytosis (Shear et al., 1989). In this work, uninfected animals exhibited a dimorphic pattern in IFN-γ levels; vehicle-treated intact males exhibited higher concentrations than females in the same condition. It is interesting to note that administering testosterone to uninfected intact animals eliminated the dimorphic pattern; in addition, gonadectomy reduced IFN-γ concentration in vehicle-treated males. Reconstituting GX mice with testosterone increased IFN-γ concentration in females (**Figure 7A**). As expected, infection increased IFN-γ levels in intact mice of both sexes. Testosterone administration decreased the concentration of this cytokine in infected intact females. However, gonadectomy did not affect the levels of this cytokine in either sex. Nevertheless, the reconstitution of GX mice with testosterone increased the concentration of IFN-γ in infected mice of both sexes compared to GX mice treated with vehicle (**Figure 7A**).

TNF-α is a critical cytokine in the elimination of *Plasmodium* (Fong et al., 1989). In addition, TNF-α has been reported to reduce the concentration of testosterone (Maggio et al., 2005). In this work, testosterone administration to intact uninfected mice increased the TNF-α levels only in intact females. Gonadectomy generated a dimorphic pattern, with uninfected males exhibiting higher levels of this cytokine than females in the same condition. The reconstitution of GX mice with testosterone increased TNF-α concentration only in females without affecting males in the same condition, which eliminated the dimorphic pattern (**Figure 7B**). Regarding TNF-α levels in infected mice, testosterone administration did not change the concentration of this cytokine; in addition, gonadectomy decreased the concentration of TNF-α in intact females and reconstitution of GX mice with testosterone restored the initial concentration (**Figure 7B**).

Additionally, we quantified IL-6 because its increase has been associated with more severe symptoms, and cerebral malaria (Pied et al., 1992). In addition, a decrease in testosterone concentration has been correlated with an increase in IL-6 concentration (Maggio et al., 2006). In this work, we detected that in uninfected mice testosterone administration or gonadectomy did not affect the concentration of this cytokine; however, GX females reconstituted with testosterone increased the concentration of IL-6 (**Figure 7C**). In the infected mice, administering testosterone increased IL-6 concentration in the intact females compared to the vehicle-treated intact group. Conversely, gonadectomy did not change IL-6 concentration in both sexes. However, the reconstitution of the GX mice with testosterone decreased IL-6 concentration in females and generated a dimorphic pattern (**Figure 7C**).

Additionally, IL-10 is the primary anti-inflammatory cytokine in malaria, and the symptomatology becomes less severe as its concentration increases (Kumar et al., 2019). In this work, the administration of testosterone to intact uninfected mice did not modify the IL-10 concentration. Gonadectomy lowered the concentration of this cytokine in both sexes, and reconstitution of GX mice with testosterone did not change the levels of this cytokine in either sex (**Figure 7D**). On the other hand, in infected mice, administration of testosterone to intact mice or gonadectomy did not change IL-10 concentration in either sex. However, reconstituting GX mice with testosterone increased IL-10 concentration in both sexes (**Figure 7D**).

### Testosterone and gonadectomy did not modify the levels of IgM or IgG in mice infected with *P. berghei* ANKA

**Figure 8:**
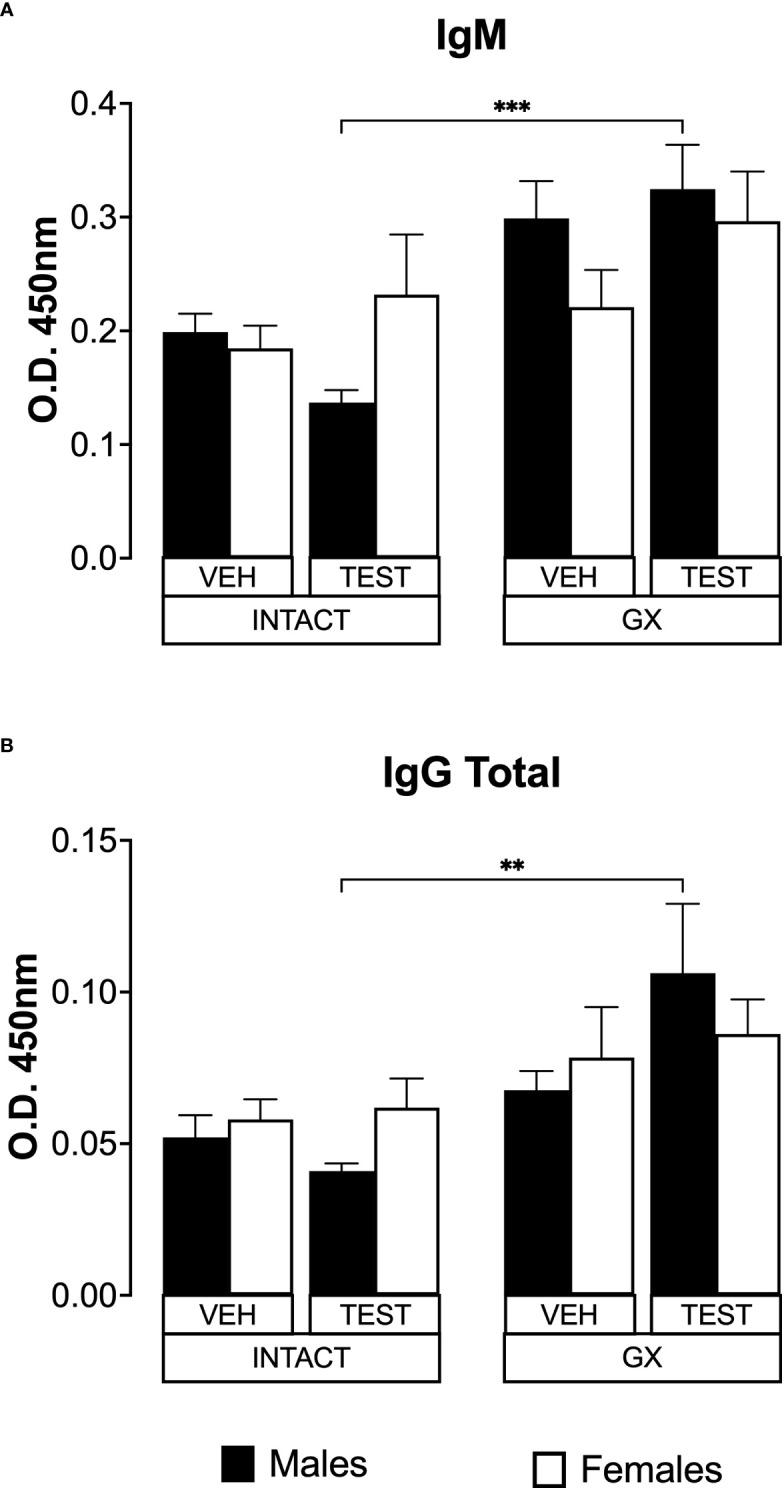
Testosterone or gonadectomy did not affect the IgM or IgG levels in mice infected with *berghei* ANKA. We used 8 groups of males and 8 groups of females, 4 groups of each sex were gonadectomised (GX), and the other 4 groups were left intact; four weeks after surgery, two groups of intact and two GX mice of each sex were administered testosterone for 3 weeks, the other two received vehicle for the same time. Finally, one intact (INTACT) and one GX group treated with testosterone of each sex were infected with *P. berghei* ANKA. The remaining groups were injected with PBS as infection controls. On day 8 post-infection, mice were sacrificed, blood was collected, and plasma used to assess the concentration of IgM and IgG. Histogram in **(A)** represents the mean ± SEM of IgM plasma levels and the histogram in **(B)** represents the mean ± SEM of total IgG plasma levels of the vehicle (VEH) or testosterone (TEST), intact (INTACT) or gonadectomised (GX) groups. Statistical differences were calculated using the Kruskal-Wallis test with a Dunn’s *post hoc* (**) represent statistical significance between groups p<0.01; and (***) p<0.001 (n = 10). The whole experiment was conducted in duplicate (n = 10).

IL-6 is a cytokine that promotes the maturation of B lymphocytes into antibody-producing cells (Sebina et al., 2017); antibodies are essential in *Plasmodium* infection by promoting their elimination *via* phagocytosis (Teo et al., 2016). In addition, testosterone reduces IgG1 and IgG2 antibody concentrations in mice infected with *Plasmodium chabaudi* (Benten et al., 1997). Therefore, in this work we studied whether modifying testosterone concentration impacted IgM and IgG antibody levels specific against *P. berghei* ANKA. Testosterone administration or gonadectomy did not modify the IgM concentration in intact male or female mice. However, testosterone-reconstituted GX mice had higher IgM concentrations than testosterone-treated intact mice (**Figure 8A**). Finally, testosterone administration or gonadectomy did not modify the concentration of total IgG in either sex, and the reconstitution of GX mice with testosterone also did not change the concentration of these antibodies. However, testosterone-treated GX mice increased IgG concentration compared to testosterone-treated intact mice (**Figure 8B**).

## Discussion

To determine the contribution of testosterone to sexual dimorphism in the immune response to malaria, we analysed the effect of modifying its concentration in male and female mice uninfected or infected with *P. berghei* ANKA. We used three strategies: the first was to administer exogenous testosterone to intact mice, the second was to eliminate the leading site of hormone synthesis by gonadectomy, and the third was to reconstitute GX mice with testosterone and, after hormone treatment, infect the mice with *P. berghei* ANKA. To our knowledge, this is the most complete study of the impact of testosterone on sexual dimorphism in malaria. We showed that infection, gonadectomy and testosterone administration generated sex-associated patterns. Testosterone administration generated dimorphic patterns in testosterone and DHEA concentration in mice infected with *P. berghei* ANKA. In addition, testosterone administration reduced parasitaemia in females, an effect reversed by gonadectomy. Testosterone also prevented the weight loss caused by *P. berghei* ANKA infection in intact females.

Interestingly, gonadectomy decreased haemoglobin concentration and temperature in uninfected mice while in infected animals, it induced the opposite. Furthermore, increasing or decreasing testosterone concentration significantly affected the immune response and generated dimorphic patterns. Testosterone-treated, uninfected, intact females showed a higher number of macrophages than males in the same condition; while uninfected, testosterone-treated males had more NK cells than uninfected females.

In addition, testosterone-treated intact infected females increased their CD3^+^ and CD8^+^ population and decreased their B cell population compared to males in the same condition. In contrast, infected intact males had an increased NK^+^ population (B220^-^CD3^-^CD16^+^CD32^+^). Additionally, testosterone decreased IFN-γ concentration exclusively in females, while gonadectomy decreased TNF-α concentration in female infected mice. Furthermore, testosterone administration increased IL-6 concentration only in intact females.

Interestingly, infection with *Plasmodium berghei* ANKA decreased free testosterone levels, particularly in females, which induced a dimorphic pattern; this finding has also been observed in mice undergoing recrudescence with *Plasmodium chabaudi* (Barthelemy et al., 2004). It would be essential to investigate whether it is the host that down-regulates testosterone concentration to reduce the immunosuppression caused by the infection and thus better contend with the parasite, or whether it is the parasite that metabolises testosterone and therefore reduces its concentration as occurs with *Taenia crassiceps* (Larralde et al., 1995).

In this work, a remarkable finding was that testosterone administration increased DHEA concentration only in infected females, probably as a negative feedback mechanism that regulates the concentration of sex steroids in females (Miller, 1988; Payne and Hales, 2004). As expected, gonadectomy decreased 17β-oestradiol levels in both sexes, since gonads are the leading organ of its synthesis. However, the reconstitution of GX mice with testosterone did not reconstitute 17β-oestradiol concentration in either sex probably due to the absence of gonads.

We also evaluated whether testosterone induced different sex patterns in the severity of infection, and we found that testosterone administration reduced parasitaemia in intact females which generated a dimorphic pattern in intact mice. In general, males are more susceptible to several parasitic diseases than females, for example to *Trypanosoma rhodesiense* (Greenblatt and Rosenstreich, 1984), *Leishmania major* (Mock and Nacy, 1988), or *Brugia pahangi* (Nakanishi et al., 1989). Our result corroborates what has been described in *Schistosoma mansoni* infection; in which administering testosterone before infection reduces parasitaemia and increases survival in female mice (Nakazawa et al., 1997). Particularly, in murine malaria, administering testosterone increases the susceptibility of male mice infected with *Plasmodium berghei* (Kamis and Ibrahim, 1989) and females infected with *Plasmodium chabaudi* (Wunderlich et al., 1991). It is likely that the decrease in parasitaemia detected in intact females is due to the conversion of some of the administered testosterone into oestrogens in the gonads. While in gonadectomized females a mechanism independent of the concentration of sex hormones is probably involved.

Additionally, because anaemia is a malaria feature, we measured haemoglobin concentration; we found that testosterone decreased haemoglobin concentration only in intact male mice; in contrast, it has been shown testosterone induces erythropoiesis and increases haemoglobin concentration (Mancera-Soto et al., 2021). A possible explanation for this discrepancy is that in our model, testosterone increased parasitaemia and thus erythrocyte destruction, which would explain the decrease in haemoglobin concentration, and differences in testosterone levels could explain the differences between infected males and infected females. Furthermore, in uninfected mice, gonadectomy decreased haemoglobin concentration, a finding possibly due to a decrease in 17β-oestradiol level, as this hormone promotes the division of haematopoietic stem cells (Nakada et al., 2014).

Testosterone administration decreased weight loss in intact females compared to males in the same condition, which generated a dimorphic pattern; a probable explanation for this finding is that testosterone increased TNF-α concentration, and this cytokine produces weight loss and proteolysis (Fong et al., 1989).

On the other hand, given that testosterone induces immunosuppression, we examined the effects of this hormone on the different immune response cells in the spleen. These cells synthesise cytokines required for the maturation and activation of other immune cells and also help for antibodies production as an essential step for the control of *Plasmodium* infection (Perez-Mazliah and Langhorne, 2014).

In uninfected, vehicle-treated females, gonadectomy increased CD3^+^ cells; since 17β-oestradiol promotes the expression of Fas ligand and the concentration of this steroid was decreased by gonadectomy, it is likely that the increase in this population is due to a reduction in apoptosis because 17β-oestradiol induces apoptosis related to Fas/FasL pathway (Yao and Hou, 2004).

Interestingly, testosterone generated a dimorphic pattern in the CD4^+^ T lymphocytes of uninfected mice; this population decreased exclusively in females, a likely explanation for this finding is that testosterone inhibits IL-12 signalling and, as a consequence, CD4^+^ T lymphocytes do not mature, as described by Kissick et al. (2014). Likewise, intact testosterone-treated infected females increased the CD3^+^ and CD3^+^CD8^+^ populations, corroborating the finding of Benten et al. in mice infected with *P. chabaudi* (Benten et al., 1993). In the present work, administering testosterone increased DHEA concentration. Therefore, it is likely that the increase in CD3^+^CD8^+^ is due to the rise in the concentration of DHEA that we detected in infected females, as described by Rasmussen et al. in *Cryptosporidium parvum* infection, because DHEA promotes the proliferation of CD8^+^ T cells (Rasmussen et al., 1995). In addition, testosterone treated and uninfected GX males decreased CD8^+^. These results contrast with those of Roden et al (Roden et al., 2004), who reported that androgen deprivation through castration increases this cell population. This discrepancy is probably due to a difference in the mouse strain, because they used genetically modified C57Bl/6 mice. In addition, an interesting finding was that the CD8^+^ population changed profoundly with infection.

Additionally, testosterone decreased the B220^+^ population in females and gonadectomized animals, generating a dimorphic pattern independent of infection. A likely explanation for this finding is that testosterone decreases the survival factor BAFF, which is essential for regulating the number of B cells in the spleen (Wilhelmson et al., 2018) consequently, it could have reduced this population in this work. Additionally, in infected mice, the decrease in the number of B220^+^ cells could be because they mature in the presence of IL-6, a process in which they lose their marker and, therefore, their number was reduced (Kunimoto et al., 1989). Testosterone increased the macrophage population in intact, uninfected females, resulting in a dimorphic pattern. Accordingly, gonadectomy decreased this population in uninfected females, eliminating the pattern. In contrast, in infected mice, gonadectomy increased the number of macrophages in both sexes; the latter finding is explained by the fact that infection activates macrophages; thus, it would be important to evaluate the effect of testosterone on macrophage phagocytic activity.

Other essential cells in the immune response to *Plasmodium* are NK cells, which produce IFN-γ that promotes the elimination of *Plasmodium via* phagocytosis (Roetynck et al., 2006). In general, it has been described that testosterone does not affect the activity of NK cells (Sorachi et al., 1993). However, in this work, we detected that testosterone administration increased the number of NK cells in intact males regardless of infection, which generated a dimorphic pattern. It is likely that in uninfected mice, the increase in these cells is explained by the rise in the concentration of DHEA, since it has already been shown that DHEA increases this population in the absence of IL-6 in mice (Zeckey et al., 2010). However, this did not happen in females in the same condition. Nonetheless, in infected mice, the increase in NK cells was exclusive to males, which could be due to the decrease in the concentration of 17β-oestradiol since it has been shown that this hormone suppresses NK cell cytotoxicity and proliferation *in vitro* NK cells (Hao et al., 2008).

Since the main complications in malaria are associated with an increase in proinflammatory cytokines, we analysed the effect of testosterone on the concentration of these molecules. We found that testosterone decreased IFN-γ levels in infected intact females. Since this hormone reduces IFN-γ production in mice (Powell and Sonnenfeld, 2006), a likely explanation for the observed decrease in IFN-γ is that DHEA increased in this group. In addition, it has been described that the increase in testosterone concentration decreases the levels of IL-6 (Zhang et al., 1998). In our study, testosterone administration increased the IL-6 levels exclusively in intact females generating a dimorphic pattern. Interestingly, this increase in IL-6 could explain the increase in CD8^+^ cells, as this cytokine has been shown to promote CD8^+^ cell proliferation.

On the other hand, testosterone reconstituted GX mice increased IFN-γ concentration in both sexes. However, they did not decrease parasitaemia, probably because this group also increased IL-10 concentration, and this cytokine negatively regulates the effects of IFN-γ (Marchant et al., 1994). In addition, testosterone has been shown to increase IL-10 concentration (Liva and Voskuhl, 2001).

This work suggests that the immunosuppressive activity of testosterone in malaria is mediated, at least in part, by IL-10, making it important to study the effects of testosterone on B lymphocytes, considering that the impact of the hormone on this cell population in malaria is unknown. In addition, we did not detect that testosterone generated statistically significant differences in IgM or IgG antibody concentration, probably because the samples were taken on day eight post-infection. This is not yet enough time for the levels to increase. However, it is noteworthy that testosterone-treated GX mice had higher IgM and IgG antibody concentrations than intact testosterone-treated mice; it has been shown that high testosterone concentrations in males reduce the production of antibodies against *P. berghei* sporozoites, thus gonadectomy would explain the increased antibody concentration (Vom Steeg et al., 2019). Another likely explanation is that 17β-oestradiol regulates the expression of genes involved in B-cell selection and activation gonadectomy by lowering 17β-oestradiol concentration would prevent B-cell precursors from being eliminated by apoptosis and thus increase the levels of antibodies (Grimaldi et al., 2002).

We are aware that a limitation in this work is that the increase of the concentration of testosterone could promote its biotransformation into oestrogens in the gonads and even extra gonadally. Therefore, we quantified 17β-oestradiol and free testosterone concentrations (the biologically active testosterone) (Wheeler, 1995). However, it would also be essential to quantify the total testosterone concentration.

## Conclusions

In this work, we showed that the response to increased testosterone concentration is different between sexes: females increased the precursor hormone DHEA that promoted IL-6 mediated immune response; males increased the concentration of testosterone, which has immunosuppressive activity. In addition, testosterone promoted an increase in CD3^+^CD8^+^ cells in females, while it increased the NK cell population in males.

**Figure 9:**
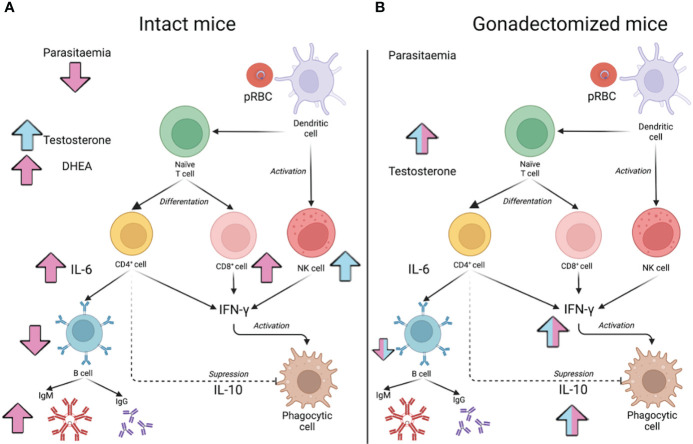
Model of testosterone involvement in sexual dimorphism of the immune response in malaria. The effect of testosterone administration on intact male or female mice is shown in **(A)** and reconstitution of gonadectomised male and female mice is shown in **(B)**. Pink arrows indicate the increase or decrease of the indicated variable in females, while in males, the effect of testosterone is indicated by blue arrows. In **(A)**, administering testosterone to intact males increased the concentration of free testosterone (Testosterone) and increased the percentage of NK cells in the spleen but did not significantly modify parasitaemia. In contrast, increasing testosterone concentration in females raised DHEA concentration, which promoted an increase in the number of CD8^+^ cells in the spleen; furthermore, it increased serum IL-6 concentration, which, in turn, could boost the maturation of B cells (B220^+^) to IgM-producing plasma cells. In contrast, increasing testosterone concentration in females increased DHEA levels, which promoted a rise in the number of CD8^+^ cells in the spleen; furthermore, it increased serum IL-6 concentration, which, in turn, could boost the maturation of B cells to IgM-producing plasma cells. However, as these cells mature, they lose their surface marker, so their number in the spleen decreases. These results would explain why parasitaemia in females was reduced. In **(B)**, the effect of testosterone reconstitution in gonadectomised mice is shown. When the effect is the same in both sexes, the arrows are bicoloured (pink and blue). Reconstituting gonadectomised mice with testosterone increased the concentration of free testosterone, and this decreased the number of B220^+^ cells and increased the concentration of IL-10, which probably reduced the IFN-γ-mediated proinflammatory response and, therefore, no effects on parasitaemia were detected.

Additionally, the immunosuppressive activity of testosterone in our malaria model is mediated, at least in part, by IL-10; this cytokine negatively regulates the proinflammatory response. The results of this work contribute to understanding the participation of this hormone in sexual dimorphism in malaria. Additionally, modify the testosterone levels affects the specific immune response against *Plasmodium* (**Figure 9**). Furthermore, our findings suggest that the immune response to pathogens can be altered in men at puberty when testosterone concentration increases, at andropause when it decreases, or in transgender individuals who are given testosterone as gender affirming hormone therapy.

## Data availability statement

The raw data supporting the findings of this paper will be made available by the authors without undue reservation.

## Ethics statement

The animal study was reviewed and approved by Care and Use of Laboratory animals adopted by the Mexican Regulation of Animal Care and maintenance (NOM-062-ZOO-1999, 2001). The protocol received approbation from the Local Ethics Committee at FES Zaragoza, UNAM (registration number 28/04/SO/3.4.1).

## Author contributions

JA-C: design of experiments, data obtention, discussion of results, and wrote the paper. LAC-C data obtention, image design, results discussion of FOB-G data processing, analysis and results discussion, OF-R quantification of cytokines and results discussion, TN-P and MSL-P data obtention and results discussion, DRC-R quantification of sex steroids and results discussion, AC-S results discussion and statistical analysis, ML-H conceived the project, designed experiments, performed analysis and discussion of results, and wrote the paper. All authors contributed to the article and approved the submitted version.

## Funding

This work was supported by PAPIIT IN220417 and IN228620, DGAPA UNAM, awarded to ML-H. JA-C awarded the fellowship 742951 from the National Council of Science and Technology of Mexico (CONACyT), this work is part of requirements for his PhD degree from the Posgrado en Ciencias Biológicas, UNAM. LC-C, OF-R, FB-G and TN-P are CONACyT fellows from the Posgrado en Ciencias Biológicas, UNAM.

## Acknowledgments

We thank Leticia Moreno-Fierros and Ana María Cevallos Gaos for the helpful discussion of the results. Additionally, we thank the animal house workers: Adriana Altamirano-Bautista, Román Hernández-Meza and Dolores Elizabeth Guzmán-Andrade, for performing gonadectomy and taking care of the animals in this study. Finally, we thank the technician Guadalupe Gómez García for her help with flow cytometry.

## Conflict of interest

The authors declare that the research was conducted in the absence of any commercial or financial relationships that could be construed as a potential conflict of interest.

## Publisher’s note

All claims expressed in this article are solely those of the authors and do not necessarily represent those of their affiliated organizations, or those of the publisher, the editors and the reviewers. Any product that may be evaluated in this article, or claim that may be made by its manufacturer, is not guaranteed or endorsed by the publisher.
